# Clinician and patient perspectives on digital technology platforms for healthcare delivery

**DOI:** 10.3389/fdgth.2026.1851786

**Published:** 2026-06-23

**Authors:** Lillian C. Chen, Mahsa Yarmohammadi, Peter P. Zandi, Melvin Ortega, Shari M. Lawson, Ore Afon, Mark Dredze, Leslie Miller

**Affiliations:** 1 Department of Gynecology and Obstetrics, Johns Hopkins University School of Medicine, Baltimore, MD, United States; 2 Center for Language and Speech Processing, Johns Hopkins University, Baltimore, MD, United States; 3 Department of Psychiatry and Behavioral Sciences, Johns Hopkins University School of Medicine, Baltimore, MD, United States; 4 Department of Obstetrics and Gynecology, Howard University College of Medicine, Washington, DC, United States; 5 Department of Computer Science, Johns Hopkins University, Baltimore, MD, United States

**Keywords:** AI, artificial intelligence, chatbot, healthcare, LLM

## Abstract

This study examines clinician and patient experiences and concerns regarding the use of digital technology platforms, particularly artificial intelligence (AI) chatbots, for clinical care. We conducted focus groups with clinicians and patients across two disciplines – young adult mental health and prenatal care – to explore these themes. While clinicians and patients both recognized the benefits of AI chatbots, they also noted the potential for misuse and inability to consider the clinical context among other concerns. We call for AI chatbots to provide clear, culturally sensitive communication, transparent attribution of sources, disclaimers for follow-up with health care professionals, and a form of regulation to avoid bias and misuse.

## Introduction

Health information chatbots, AI driven agents that deliver information through digital conversational interfaces, have significantly proliferated in recent years. The adoption of AI chatbots was catapulted by the COVID-19 pandemic, during which remote assessment was emphasized. While the utilization of digital technology platforms, particularly AI chatbots, has become widespread, there is concern about the accuracy of information, possible omission of critical information, and acceptability of these tools in clinical practice (1–3). There is limited evidence directly comparing clinician and patient perspectives on the integration and dissemination of health care oriented AI chatbots. We explored these elements from the perspectives of practicing clinicians and their patients in two different disciplines – young adult mental health and prenatal care – to identify factors that should be considered when evaluating the acceptability of AI chatbots generating clinical advice.

## Methods

We conducted a series of semi-structured focus groups with practicing clinicians in psychiatry and obstetrics and gynecology at two academic medical institutions and additional semi-structured focus groups with patients receiving mental health care or prenatal care. Providers and patients were recruited via convenience sampling. Providers included attending physicians, resident and fellow physician trainees, and advanced practice providers within the fields of psychiatry and obstetrics and gynecology. Patients included individuals who currently or previously received mental health or prenatal care within our health system and were over the age of 18. Focus groups were conducted by research team members with experience leading semi-structured focus groups. Each focus group queried participants about the acceptability and concerns regarding digital technology platforms more broadly before focusing specifically on experiences with AI chatbots with specific prompts tailored towards clinicians and patients on their experiences using AI chatbots for clinical and non-clinical tasks. The focus groups were audio recorded and transcribed without identifying factors to ensure participant anonymity. Three study team members reviewed the transcripts to identify response themes using thematic analysis coding. This study was approved by the Johns Hopkins University Institutional Review Board.

## Results

Seven focus groups with mental health and obstetrics and gynecology providers including 44 total clinicians were conducted between December 2024 and January 2025. Clinicians included 31 physicians and 12 advanced practice providers. Individual focus groups with eight patients receiving mental health or prenatal care were conducted between February and March 2025. Patients included 5 female, 2 male, and 1 non-binary patient and all received mental health or prenatal care within our hospital system within the last calendar year.

Clinicians and patients acknowledged the growing complexities of navigating multiple information sources and the evolving expectations around healthcare delivery. Patients in both disciplines reported frequently seeking health information through a variety of online sources. For prenatal care, patients commonly inquired about medication and exercise safety, dietary choices, and hospital procedures. For mental health, patients sought answers to treatment of mood disorders and substance use, medication side effects, and how to navigate challenging relationships. These concerns are often personal and urgent, prompting patients to consult the most accessible sources, such as search engines and social media. Patients reported that they often turned to these digital platforms as they provided quicker responses than approaching their healthcare teams.

When asked specifically about AI chatbots, however, the patients interviewed expressed discomfort and skepticism. Patients reported the lack of human empathy and the potential for inaccuracy as drawbacks, in contrast to content on social media that they perceived as more relatable. One patient cited that he felt unsure about AI chatbots because “I don't entirely know what sources it's pulling from. I know it can give me the references for that, but I’ve also seen it make up stuff a lot.” Some patients also cited the benefit of being able to flag inaccurate content on social media while AI chatbots did not offer this option. While one patient noted positive experiences using AI chatbots for writing tasks, this confidence did not extend to medical use. Other patients cited concerns regarding information security, stating “I know the data is being stored somewhere. I don't know if that's being used for the algorithm itself, or how it's being used. Or if that is then tied to my own IP address or my own data.”

Clinicians, on the other hand, frequently cited concerns about the quality and interpretation of online information when asked broadly about their experiences with digital technology platforms. Clinicians reported that patients often come to appointments with preconceived notions or self-diagnoses based on anecdotal content from the internet or social media. Additionally, patients with introductory medical knowledge may assume greater expertise than they possess, creating friction in the provider-patient relationship. Clinicians expressed concerns about misinformation leading to unrealistic expectations regarding pregnancy care and mental health interventions. Confirmation bias further complicates this, as individuals may selectively absorb information that validates their assumptions. To combat these risks, clinicians recommend reputable sources such as the American Academy of Child and Adolescent Psychiatry (AACAP), National Alliance on Mental Illness (NAMI), the Child Mind Institute for mental health references, the American College of Obstetricians and Gynecologists (ACOG) and the Center for Disease Control (CDC) for prenatal health references.

When queried about AI chatbots specifically, clinicians recognized the potential for AI chatbots to improve access to basic information in an expeditious manner, for example listing foods to avoid during pregnancy or examples of conversation starters for socially anxious young adults. However, they had significant concerns around their use for more complex or detailed medical issues. For example, one clinician cited that “having contractions at 22 weeks is very different than having contractions at 36 weeks, so unless there is a way to make sure answered were nuanced, it would be hard for me to trust what is coming from them [AI chatbots].” Clinicians emphasized the need for AI chatbots to present clear, accurate information, include disclaimers encouraging professional consultation when appropriate, and safeguards to maintain privacy and data security standards, particularly when navigating high-risk situations such as self-harm, substance use, or pregnancy complications. Several clinicians suggested that AI chatbots should be governed by regulatory frameworks to ensure accountability. Moreover, the potential misuse of chatbots as a replacement for professional care raises concerns about delaying timely medical attention and undermining trust in the healthcare system. Finally, clinicians expressed concern regarding legal liability if AI chatbots were implemented as part of their clinical practice to respond to patient inquiries. Overall, clinicians felt that AI chatbots should serve as educational tools and guide users towards reputable resources. However, for individualized concerns, direct physician interaction remains irreplaceable.

## Discussion

This qualitative study of clinician and patient experiences with AI chatbots providing healthcare information highlights the benefit of expeditious dissemination of information. There is concern, however, regarding the delivery of accurate and complete information, particularly if the clinical context can impact recommendations. Overall, clinicians and patients agreed on the following best practices for AI chatbot integration into healthcare delivery: clear, culturally sensitive communication with use of peer-reviewed, evidence-based sources, disclaimers encouraging follow-up with healthcare professionals when warranted, and the creation of a regulatory oversight system in the future to avoid bias and misuse. Our study uniquely compares the clinician and patient experience using the same subset of guiding questions. Our results are congruent with prior studies that found similar concerns for use of digital chatbots in various clinical disciplines (4–8). Limitations of this study include the recruitment of participants at two medical centers in two specific disciplines who may have different experiences compared to their peers providing and receiving care in other disciplines or practice settings. Additionally, we also note the limited sample size of patients included in this study compared to clinicians, which may not reflect the overall patient experience. Furthermore, this qualitative study cannot assess differences in outcomes attributed directly to the use of AI chatbots. These findings suggest a framework for potential issues to consider when integrating AI chatbots into clinical practice and highlights end-user concerns for AI chatbot developers to address when designing these powerful tools.

## Data Availability

The raw data supporting the conclusions of this article will be made available by the authors, without undue reservation.
